# Opportunity for early de-escalation of antibiotics in Enterobacterales bacteremia with multiplex PCR rapid diagnostic technology

**DOI:** 10.1017/ash.2024.488

**Published:** 2025-01-27

**Authors:** Erin Deja, Adam Greenfield, Sangeeta Sastry, Barry Rittmann

**Affiliations:** 1 Department of Pharmacy, Virginia Commonwealth University Health System, Richmond, VA, USA; 2 Department of Internal Medicine, Division of Infectious Diseases, Virginia Commonwealth University Health System, Richmond, VA, USA

## Abstract

Gram-negative bacteremia and antimicrobial resistance cause significant morbidity and mortality. Strategies are needed to optimize rapid and effective antimicrobial therapy with early de-escalation. In this brief, we describe the utilization of multiplex polymerase chain reaction rapid diagnostic technology for the early de-escalation of broad-spectrum antimicrobials in patients with Enterobacterales bacteremia.

## Introduction

Bloodstream infections are associated with significant morbidity and mortality, and timely administration of effective antibiotics greatly affects outcomes.^1^ Sepsis guidelines recommend empiric broad-spectrum antibiotics to increase the likelihood of activity against identified pathogens.^2^ Unfortunately, antibiotic overuse is associated with mounting antimicrobial resistance. Over 2.8 million antimicrobial-resistant infections and 35,000 associated deaths occur annually in the United States.^3^ Conventional culture-based techniques take days to identify causative organisms, delaying de-escalation to optimal, narrow-spectrum antimicrobials. Clinicians require alternative diagnostics to guide early antibiotic de-escalation for bacteremia.

Rapid microbiology diagnostics such as multiplex polymerase chain reaction (PCR) decrease the time to diagnosis for various infections. The use of rapid diagnostics for bacteremia with antimicrobial stewardship program (ASP) intervention is associated with improvement in time to appropriate antibiotics, hospital length of stay, and cost.^4–7^ BioFire® FilmArray Blood Culture Identification 2 (BCID2) identifies 33 organisms and 10 antimicrobial resistance genes, including CTX-M, the most common extended-spectrum β-lactamase (ESBL).^8^ Although BCID2 reasonably predicts gram-positive resistance via mecA/C and vanA/B, mechanisms of gram-negative resistance are more diverse and may not be apparent on BCID2 (eg, TEM/SHV ESBLs, efflux pumps, porin mutations).

Virginia Commonwealth University Health System (VCUHS) implemented BCID2 in March 2023 and established optimal treatment recommendations for all results based on the organism identified, presence or lack of resistance genes, and local resistance patterns. ASP pharmacists receive results in real time, critically assess each patient, and provide treatment recommendations. We measured the congruence between BCID2 resistance gene detection and routine susceptibility testing and validated the adequacy of VCUHS BCID2 treatment recommendations for Enterobacterales bacteremia.

## Methods

BCID2 results for Enterobacterales species from March 12 to June 19, 2023, were retrospectively identified for patients at VCUHS Medical Center, an 800+ bed level 1 trauma center, and 2 VCU-affiliated community hospitals. Enterobacterales identified by BCID2 include *Enterobacter cloacae* complex, *Escherichia coli*, *Klebsiella aerogenes*, *Klebsiella oxytoca*, *Klebsiella pneumoniae*, *Proteus* spp., *Salmonella* spp., and *Serratia marcescens*. BCID2 reports species within the Enterobacterales family but without a specific target as “Enterobacterales (unspecified).” *K. aerogenes* and *E. cloacae* complex were excluded due to their high risk for clinically significant inducible AmpC β-lactamase production. Blood cultures were incubated in the BACTEC system (Becton Dickinson, Sparks, MD). Upon signaling a positive culture, Gram stain and BCID2 (bioMerieux, Marcy l’Etoile, France) were performed. Only the first positive blood culture was tested on BCID2 in patients with persistently positive cultures with identical Gram stain morphology. Colonies isolated from blood-agar subcultures were identified using Bruker Biotyper matrix-assisted laser desorption/ionization time-of-flight (MALDI-TOF) mass spectrometry (Bruker, Bremen, Germany). Antimicrobial susceptibility testing was performed by disk diffusion and interpreted according to Clinical & Laboratory Standards Institute (CLSI) breakpoints (CLSI M100-Ed34, 2023). BCID2 results were reviewed for agreement with final cultures and susceptibility to ceftriaxone, cefepime, piperacillin/tazobactam, and meropenem.

## Results

A total of 270 results were included. The most identified organism was *E. coli* (51.5%) (Table 1). Organisms identified on BCID2 were concordant with final culture results in all but 2 polymicrobial samples, which detected only *E. coli* on BCID2 but grew *K. pneumoniae* and coagulase-negative *Staphylococcus* spp. on routine culture, respectively. Eighteen isolates were unspecified Enterobacterales, of which one was *Citrobacter freundii*, an inducible AmpC producer later identified by MALDI-TOF. Susceptibility results are summarized in Table 2. Twenty-seven (10%) isolates were CTX-M positive and all resistant to ceftriaxone. One isolate (0.4%) was Klebsiella pnuemoniae carbapenemase (KPC) positive and resistant to meropenem. The remaining 242 isolates were negative for all resistance genes detected by BCID2. Of these, 3 (1.2%) were not susceptible to ceftriaxone (zone diameters = 22 mm [intermediate], 18 mm [resistant], 15mm [resistant]). All 3 isolates were susceptible to cefepime and meropenem but had variable susceptibility to piperacillin/tazobactam. Notably, 8 (3.3%) CTX-M negative isolates were resistant to piperacillin/tazobactam, most of which were *K. pneumoniae*. An additional 24 (9.9%) isolates tested susceptible dose-dependent (S-DD) to piperacillin/tazobactam. Cefepime and meropenem maintained activity against all isolates negative for resistance genes.

**Table 1.** Included Enterobacterales identified by BCID2

Organism	N
*Escherichia coli*	139 (51.5%)
*Klebsiella pneumoniae*	74 (27.4%)
*Serratia marcescens*	16 (5.9%)
*Klebsiella oxytoca*	10 (3.7%)
*Proteus* spp.	8 (3%)
*Salmonella* spp.	5 (1.9%)
Enterobacterales, unspecified^1^	18 (6.7%)

Note. BCID2, BioFire® FilmArray Blood Culture Identification 2.

1 *Citrobacter freundii (1), Citrobacter koseri (4), Pluralibacter* spp. *(1), Providencia* spp. *(4), Lelliottia amnigena (1), Morganella* spp. *(2), Pantoea* spp. *(5).*

**Table 2.** Selected susceptibilities by resistance gene detected

	Ceftriaxone	Cefepime	PTZ**^1^**	Meropenem
**None (n=242)**	98.8%	100%	96.7%	100%
**CTX-M (n=27)**	0%	29.6%^2^	88.9%^2^	96.4%^3^
**KPC (n=1)**	0%	0%	0%	0%

1 PTZ: piperacillin/tazobactam.

2 Includes susceptible dose-dependent isolates.

3 One isolate positive for both CTX-M and KPC.

## Discussion

In this study, CTX-M detection was 90% sensitive and 100% specific for predicting ceftriaxone resistance in Enterobacterales. VCUHS 2023 antibiogram data indicate ceftriaxone susceptibility for 90.4% of non-inducible AmpC-producing Enterobacterales. Our ASP recommends ceftriaxone for most Enterobacterales bacteremia without BCID2-identified resistance genes. However, providers are often reluctant to de-escalate without confirmed susceptibility, as there are multiple potential mechanisms of gram-negative antimicrobial resistance. Production of a β-lactamase is the most common in Enterobacterales, but nearly 3,000 unique enzymes exist. Although CTX-M accounts for >90% of ESBL-producing organisms in the United States, there are other enzymes BCID2 cannot detect that cause ceftriaxone resistance.^8^ Interestingly, in this study, CTX-M negative isolates were more often susceptible to ceftriaxone than piperacillin/tazobactam, which is commonly used as empiric gram-negative therapy at VCUHS. Approximately 10% of isolates tested piperacillin/tazobactam S-DD, which requires treatment with high-dose extended infusion to achieve adequate time above the minimum inhibitory concentration (MIC). This pattern may reflect selective pressure due to piperacillin/tazobactam overuse and increases the risk for inadvertent selection of inappropriate empiric antibiotics.

We add to the body of literature on the use of multiplex PCR in management of gram-negative bacteremia. A retrospective, observational study in India (n = 200; 183 of 200 gram-negative isolates) reported 97% concordance between BCID2 resistance gene detection and phenotypic drug susceptibility. The 6 discordant isolates were in polymicrobial cultures.^9^ A prospective, single-center study in Korea (n = 117; 64 of 117 gram-negative isolates) found BCID2 accurately predicted phenotypic susceptibility for 96.9% of gram-negative isolates. Two strains of ESBL *Serratia marcescens* were not identified as CTX-M positive.^10^ Another prospective, single-center study in Germany (n = 180; 93 of 180 gram-negative isolates) found 16 gram-negative isolates resistant to third-generation cephalosporins, but only 12 identified as CTX-M positive by BCID2. Three of the CTX-M negative isolates carried *bla*_SHV_ and/or *bla*_TEM_. The resistance mechanism of the fourth isolate was not determined.^11^ Finally, a prospective, multicenter study in France (n = 152; 98 of 152 gram-negative isolates) found 2 false-positive CTX-M and 2 ESBL isolates of *E. cloacae* not identified as CTX-M positive by BCID2.^12^

Our study has several limitations. Some Enterobacterales, such as *Citrobacter freundii*, *Hafnia alvei*, and *Yersinia enterocolitica*, are not identified at the genus or species level by BCID2. Due to the risk of inducible AmpC resistance, ceftriaxone should be avoided in these isolates. Furthermore, BCID2 does not identify constitutive AmpC production. The 3 ceftriaxone-resistant isolates in this study remained susceptible to cefepime and meropenem but were variably susceptible to piperacillin/tazobactam, a phenotype possibly suggestive of AmpC production. Finally, these data may not be generalized to institutions with differing patient populations or antibiograms, such as locations with higher prevalence of non-CTX-M ESBLs.

Treatment of sepsis using antibiotics with local susceptibility ≥90% results in decreased in-hospital mortality. This threshold is often used as a benchmark for selection of appropriate empiric therapy.^13^ In our study, ceftriaxone demonstrated 98.8% susceptibility for Enterobacterales negative for resistance genes on BCID2. These data suggest that early de-escalation to ceftriaxone in patients with Enterobacterales bacteremia without resistance genes identified by BCID2 may be appropriate. Providers must still assess patients individually for severity of infection, suspected source, and risk for polymicrobial infection. In patients at high risk for drug-resistant pathogens, infectious diseases and/or ASP-trained individuals should be involved in the interpretation of PCR results and subsequent treatment recommendations. We highlight an opportunity for safe and effective early de-escalation of antibiotics for the treatment of Enterobacterales bacteremia in appropriate patient populations.
